# Cocoa Shell Infusion: A Promising Application for Added-Value Beverages Based on Cocoa’s Production Coproducts

**DOI:** 10.3390/foods12132442

**Published:** 2023-06-21

**Authors:** Johannes Delgado-Ospina, Luigi Esposito, Junior Bernardo Molina-Hernandez, José Ángel Pérez-Álvarez, Maria Martuscelli, Clemencia Chaves-López

**Affiliations:** 1 Department of Bioscience and Technology for Food, Agriculture and Environment, University of Teramo, Via R. Balzarini 1, 64100 Teramo, Italy; lesposito2@unite.it (L.E.); jbmolinahernandez@unite.it (J.B.M.-H.); cchaveslopez@unite.it (C.C.-L.); 2 Grupo de Investigación Biotecnología, Facultad de Ingeniería, Universidad de San Buenaventura Cali, Carrera 122 # 6-65, Cali 76001, Colombia; 3 IPOA Research Group, Agro-Food Technology Department, Higher Polytechnic School of Orihuela, Miguel Hernández University, CYTED-Healthy Meat. 119RT0568 “Productos Cárnicos más Saludables”, 03312 Orihuela, Spain; ja.perez@umh.es

**Keywords:** antioxidants, sensory analysis, volatile compounds, cocoa shell, food waste

## Abstract

The cocoa shell (CS) is being incorporated into different food products due to its recognized content of bioactive compounds. In the case of cocoa shell infusions (CSI), the bioactive compounds that manage to be transferred to the infusion have yet to be clearly known, i.e., what is really available to the consumer. In this study, CS was obtained from toasted Colombian Criollo cocoa beans. Three particle sizes (A: >710 µm; B: >425 and <710 µm; C: <425 µm) were evaluated in the CSI, which was traditionally prepared by adding CS to hot water (1%). The decrease in particle size increased the antioxidant capacity (DPPH and ABTS) and the total phenolic compounds. A significant effect (*p* < 0.05) both of the particle size and of the temperature of tasting was found on some sensory attributes: greater bitterness, acidity, and astringency were due to the greater presence of epicatechin, melanoidins, and proanthocyanidins in the smaller particle sizes. The analysis of the volatile organic compounds showed that the CSI aroma was characterized by the presence of nonanal, 2-nonanone, tetramethylpyrazine, α-limonene, and linalool, which present few variations among the particle sizes. Moreover, analysis of biogenic amines, ochratoxin A, and microbial load showed that CSI is not a risk to public health. Reducing particle size becomes an important step to valorize the functional properties of CS and increase the quality of CSI.

## 1. Introduction

The sustainability of food production caused by climate change faces different challenges in the future for the food industry and consumers. The industry needs to produce healthy and wellness foods to improve consumers’ welfare. These challenges must be met with transformations in how food is manufactured and consumed to meet the needs of a constantly growing population while reducing, at the same time, its environmental impact.

Responsible production and consumption are one of the objectives of the 2030 Agenda for Sustainable Development, which promotes the use of by-products/co-products to avoid/reduce losses during food production. In this context, the use of plant-based by-products is an opportunity to discover and add value to the biomolecules found in the inedible or underutilized parts of vegetables, so it is necessary to research their benefits and find a way to incorporate them through processed food and then into the diet [1].

Cocoa is an agricultural commodity of great importance worldwide due to its versatility to be incorporated into multiple food and cosmetic products. Its commercialization, including processed products, represented more than 57 billion dollars ($57,180,986,000) for exporting countries in 2021, growing by 12% compared with the previous year [2]. Within this panorama, the commercialization of cocoa shells, husks, skins, and other cocoa coproduct waste represented more than 40 million dollars ($40,234,000) for exporting countries in 2021, growing by 17% compared with the previous year [2]. It is clear that the use of cocoa residues, especially cocoa shells, has had a significant impact on the economy.

There are three varieties of cocoa: Criollo, Forastero, and Trinitario. Criollo (*Theobroma cacao* L. cv Criollo) is a cultivar from Colombia and other Latin American countries. Due to its high quality, it was declared by the International Cocoa Organization (ICCO) as “fine” and rich of “flavour”. Additionally, this cocoa shell contains bioactive compounds of interest to the food industry [1].

The cocoa shell has a high nutritional value due to its content of proteins, lipids, fiber, phenolic compounds, and theobromine, among others [1,3]. Its addition to different products has been investigated: for its contribution of biomolecules in wheat bread [4], hamburger meat [3], and enriched chocolates [5]; as a fat replacer in chocolate muffins [6] and beef burger [7]; as a wheat flour replacer on some of the chocolate cake [8]; as an antioxidant in functional biscuits [9] and olive oil jam [10].

The use of cocoa shells as an infusion is the most popular due to their flavor and ease of preparation. In some countries, it is already sold loose or in tea bags. However, there are few studies on this use. Some studies have been carried out to determine the antioxidant properties of the infusion. Still, they have been carried out with different extraction methods than those used by consumers (for example, extracted with methanol: hot water for 1 h) [11], so the components of the final infusion may be overestimated. Recent studies have been carried out to evaluate the beverage’s phenolic compounds according to particle size, extraction methods [12], and bioaccessibility [13]. However, there are still many gaps in determining all the functional characteristics of the beverage.

Since very few studies indicate the functional characteristics of the cocoa shell infusion, in this work we focus on evaluating the microbiological characteristics, volatile organic compounds, polyphenols, minerals, etc. The infusion represents both a safe beverage and an opportunity to consume biomolecules of interest for health.

## 2. Materials and Methods

### 2.1. Cacao Shell (CS)

The cacao shell (CS) was obtained from samples of cacao *Criollo* from Colombia after roasting at 135 °C for 15 min. The CS was obtained after mechanically separating the nibs. Subsequently, CS was ground in an impact mill (IKA MF 10.2, Staufen, Germany) and passed through two sieves (Sieve Standard numbers 25 and 40) to obtain three particle sizes: CS-A (>710 µm); CS-B (>425 µm and <710 µm); and CS-C (<425 µm). The proximate composition, pH, water activity (a_w_), and color were determined in CS samples in a previous study by Delgado-Ospina et al. [1].

### 2.2. Preparation of the Cocoa Shell Infusion (CSI)

For each particle size (CSI-A, CSI-B, and CSI-C), 1.0 g of the cocoa shell was added to 100 mL of water at 96 °C (boiling point) and stirred for 1 min. Then, the cocoa shell infusions were decanted for 5 min, and the supernatants were used to conduct the different analyses.

### 2.3. pH and Color Analyses

The pH was measured directly on CSI samples with an electrode probe connected to a pHmeter (model 3510, Jenway, Stone, UK).

A colorimetric analysis was performed using a CM-700d spectrophotometer (Konica Minolta, Osaka, Japan), depositing the samples in a cylindrical cell of path length 50 mm with the following settings: illuminant D_65_, observer 10°. The CIEL*a*b* color space was used. The following color coordinates were determined: Lightness (L*), red/green (+/−) co-ordinate (a*), and (+/−) yellow/blue co-ordinate (b*), hue angle *h*° (or h*_ab_), Equation (1)), color difference ΔE* (Equation (2)) were determined in CSI-B and CSI-C, taking as reference CSI-A and the reflectance spectra (wavelengths 360–740 nm).

(1) $$h{}^{\circ}=\arctan\frac{b^{*}}{a^{*}}$$

(2) $${\Delta E}^{*}=\sqrt{{\left({\Delta L}^{*}\right)}^{2}+{\left({\Delta a}^{*}\right)}^{2}+{\left({\Delta b}^{*}\right)}^{2}}$$

### 2.4. Minerals’ Determination

Minerals analyses were performed in a microwave digestor (Bergof mod. Speedwave 2) coupled with an inductively coupled plasma for the reading of the results (ICP-OES, Perkin Elmer mod. Avio200) according to the protocol given by the instrument producers and the methodology reported by Martuscelli et al. [14].

### 2.5. Microbiological Analyses

For CS, 10 g of each particle size were homogenized in a Stomacher lab blender in a 90 mL sterile saline solution. The CSI was used directly. Decimal dilutions of both suspensions (CS and CSI recently prepared) were plated and incubated according to [15]: for determined lactic acid bacteria (LAB) in MRS agar (Oxoid, Basingstoke, UK) at 37 °C in anaerobiosis for 48 h, acetic acid bacteria (AAB) in GYC agar (20 g/L glucose, 10 g/L yeast extract, 3 g/L CaCO_3_, 15 g/L agar, 70 g/L ethanol, and 250 mg/L of fluconazole) at 30 °C for 72 h, Yeasts in YPD agar (Biolife, Italiana, Milan, Italy) added with 150 ppm chloramphenicol (Sigma-Aldrich, IT, Milan, Italy) at 30 °C for 24 h; molds in DG18 Agar (Oxoid) added with 150 ppm chloramphenicol at 30 °C for 96 h; total coliforms in Violet Red Bile Glucose Agar (Oxoid, Basingstoke, UK) at 37 °C in anaerobiosis for 48 h. Mesophilic aerobic bacteria in plate count agar (PCA) at 30 °C for 48 h. The visible colony count at the end of the incubation period and the dilution factor were used to determine the number of microorganisms in the sample.

### 2.6. Determination of Ochratoxin A (OTA) from Cocoa Shells and Cocoa Shell Infusions

#### 2.6.1. Extraction of OTA from Cocoa Shells

Ochratoxin A was measured using an ochratoxin A ELISA kit (Helica biosystem, Santa Ana, CA, USA) according to the method described by Chen et al. [16]. Two grams of each particle size (CS-A, CS-B, and CS-C) of cocoa shell were extracted with 10 mL of methanol (70%) using an orbital shaker (Centomat BS -T, B.Braun, Milan, Italy) at a speed of 250 rpm for 3 min. The sample was centrifuged at 13,000× *g* rpm for 5 min, and the supernatant was collected. The sample extracted (100 uL) was diluted (1:10) and used for measurement, following the manufacturer’s instructions.

#### 2.6.2. Ochratoxin A from Cocoa Shell Infusions

After decantation, the supernatants obtained from the cocoa shell were used for the determination of ochratoxin A. The extraction was performed according to the instructions of the ochratoxin A assay kit.

#### 2.6.3. Risk Characterization

The exposure assessment for ochratoxin A in cocoa infusions was calculated as Margin of Exposure (MoE) using the benchmark dose lower confidence limit for extra cancer risk of 10% (BMDL_10_), according to Equations (3) and (4).

(3) $$\mathrm{MoE}=\frac{\mathrm{BMDL}10}{\mathrm{PDI}}$$

(4) $$\mathrm{PDI}=\frac{\mathrm{Ci}\;\times \;\mathrm{Cm}}{\mathrm{bw}}$$

where: BDML_10_ for OTA is 14.5 μg kg^−1^ bw day according to EFSA, Panel on Contaminants in the Food Chain [17], PDI is the probable daily intake of an individual (μg kg^−1^ bw day), Ci is the estimated average dose of infusion intake per day (4 oz or 0.1189 L day^−1^), Cm is the average concentration of OTA in the infusion tested (µg L^−1^), and bw is the body weight of an adult person (70 kg).

### 2.7. Biogenic Amine Determination

CSI was subjected to the biogenic amines (BAs) determination. An aliquot of each sample (0.5 mL) was mixed with 150 μL of a saturated NaHCO_3_ solution, and the pH was adjusted to 11.5 with 0.1 N NaOH. After this, 2 mL of acetone solution containing 20 mg of Dansyl chloride (Fluka) was added to the alkaline amine extract [18]. Derivatization was completed according to the protocol established by Esposito et al. [19] without modifications.

BAs detection, identification, and quantification were performed by high-performance liquid chromatography (HPLC) using an Agilent 1200 Series (Agilent Technologies, Milan, Italy), according to the method described by Esposito et al. [19] with some modifications. The separation of the analytes was carried out using a Waters Spherisorb C18 S3ODS-2 column (3 μm particle size, 150 mm × 4). Identification and quantification of cadaverine, dopamine, salsolinol, ethylamine, histamine, 2-phenylethylamine, putrescine, serotonin, spermidine, spermine, and tyramine were performed by comparing retention times and calibration curves of pure standards.

### 2.8. Antioxidant Capacity Assays on the CSI Samples

#### 2.8.1. ABTS Radical Cation (ABTS•+) Scavenging Activity and DPPH Radical Scavenging Ability Assays

The radical scavenging activity was measured by the ABTS radical cation discoloration assay, and the radical scavenging ability of the supernatants was measured using the stable radical DPPH [15]. Absorbance was determined at 734 and 595 nm, respectively, using a spectrophotometer (Genesys 10 UV, Thermo Electron, Waltham, MA, USA).

ABTS was calculated by dividing the ratio of the correlation coefficient of the dose-response curve of the sample by the correlation coefficient of the dose-response curve of the Trolox standard. DPPH was calculated by comparing the absorbance of the sample with the calibration curve of Trolox. In both cases, the results were expressed as µmol of Trolox equivalent per 100 mL of infusion.

#### 2.8.2. Total Phenolic Compounds

The Total Phenolic Compounds (TPC) used the Folin-Ciocalteu method with some modifications [15]. Absorbance was determined at 760 nm using a spectrophotometer. The results were compared with the calibration curve of gallic acid and expressed as mg of gallic acid equivalent per 100 mL of infusion.

### 2.9. Polyphenol Determination

The analysis of the phenolic pattern was carried out on CSI samples according to the method described by Martuscelli et al. [14]

The identification and further quantification of phenolic acids were conducted by means of HPLC-DAD analysis (mod. HPLC: AGILENT series 1200 Agilent Technologies, Milan, Italy). A reverse-phase C18 column (5 µ 100 Å 250 × 4.6 mm, Phenomenex Kinetex, Bologna, Italy) was used. The solvent phases utilized were water and formic acid 5% (A) and methanol (B). The flux was fixed at 1 mL/min. Data for (+) catechin, (−) epicatechin, quercitin, rutin, p-coumaric, caffeic, vanillic, chlorogenic, protocatecuic, p-hydroxide benzoic, siringic, ferulic, cinnamic, and gallic acids were spectrophotometrically read at 260, 280, and 330 nm. The quantification of the founded species was determined using calibration curves in a range going from 3 to 100 mg L^−1^.

### 2.10. Organic Acids, Sugar, and Alcohol Analyses

Fermentable sugars (glucose, sucrose, and fructose), alcohols (ethanol and glycerol), and organic acids (acetic acid, lactic acid, citric acid, and succinic acid) were analyzed with HPLC equipment (Elite Lachrom, Hitachi, Tokyo, Japan) coupled with the refractive index detector L-2400. The samples of the recently prepared cocoa shell infusion were centrifuged at 8000× *g* rpm for 10 min at 4 °C; the supernatant was filtered through 0.22 µm PVDF membranes, and 20 µL were injected into the analytical column (Supelco Gel C-610H, (300 × 7.8 mm) using 0.1% H_3_PO_4_ as the mobile phase, operating at a flow rate of 0.5 mL/min at 30 °C. The compounds were identified by comparing the retention times with the standards. They were quantified using the respective calibration curves.

### 2.11. Determination of Volatile Organic Compounds (VOCs) for HSGC-MS Analysis

Volatile compounds in the CSI samples were determined by Solid Phase Micro-Extraction coupled with Gas Chromatography Mass Spectrometry (SPME/GC-MS) as reported by [20] with some modifications. The fiber used for SPME was coated with a Divinilbenzene/Carboxen/Polydimethylxilosane (DIV/CAR/PDMS) 50/30 μm thickness. For VOC extraction, 5.0 g of CS were deposited in a 50 mL vial and heated at 50 °C in a water bath for 40 min to reach equilibrium and for another 30 min for fiber absorption. For infusion, 20 mL of sample was deposited and kept under constant agitation with identical time and temperature conditions.

Volatile peak identification was carried out by computer matching of mass spectral data with those of the compounds contained in the Agilent Hewlett-Packard NIST 98 and Wiley version 6 mass spectral databases. The volatile compound content was expressed as a relative percentage.

### 2.12. Sensory Evaluation

To evaluate sensory properties of CSI, a Quantitative Descriptive Analysis (QDA) was developed. The main objective was to identify typical descriptors for infusions. Tasters were voluntarily taking part and informed about all the possible risks to which they may be exposed that are anyway minimized; thus, ten participants were selected after a screening test of their sensory abilities. Moreover, all participants were asked about any food allergy\intolerance and informed about the aim of the research. Informative consent was given, explaining that the participation was voluntary and no penalties were forecasted for eventual withdrawal. Finally, ethical treatment of the collected data was assured in accordance with current European rules.

Several sessions of training were then run to familiarize participants with descriptors and assess odors, colors, and tastes with intensity scales. Scales used were labeled with numbers from 0 to 5, where 0 represented the absence of the attribute and 5 the maximum intensity. A total of ten hours of training split into 5 different days was enough. Evaluations were non-continuative in time; each session could last no more than 30 min and be repeated after another 30 min of breaks. This allowed for the development of an agreed-upon vocabulary for the proper and common evaluation of products tested and the building of a group of trained panelists [21,22]. All training sessions and sensory analysis were organized in the sensory laboratory of the Department of Biosciences of the University of Teramo, which meets the standards set by [23]. All activities were conducted by the panel leader. The group had ten trained panelists (6 women and 4 men) from 25 to 52 years old (mean age 31.7 ± 9.15).

Infusions of the different sizes were tasted at two temperatures: hot (55 ± 2 °C) and cold (12 ± 2 °C), for a total of six samples.

Individual white plastic cups were filled with ~30 mL of infusion, randomly numbered, and served to panelists in two separate triplets.

Panelists were asked to evaluate three classes of descriptors: color, odor, and taste. Infusions were served with any addition of sugar, honey, other sweeteners, or ingredients. Infusions were obtained by following the method of Huan Li et al. [24], with some differences. Boiling water (boiled for 1 min) was directly poured onto CS in glass pot at controlled temperature. The ratio used was 1:100 (CS:water). CS was maintained for 5 min, and liquids were filtrated. Hot samples were set in plastic cups and served. Cold samples were equally obtained and refrigerated until reaching the desired temperature.

### 2.13. Statistical Analysis

Each determination was made three times. Calculations were made for means and relative standard deviations. The least significant differences (LSD) test was used to distinguish between mean differences to examine the significance of the impacts of the factor variables (particle size, temperature of tasting).

XLSTAT software for Microsoft Excel (Addinsoft, New York, NY, USA) version 2019.1 was used to statistically analyze the data. At *p* < 0.05, all results were deemed statistically significant.

Multivariate descriptive analysis was used to understand the presence of the main descriptors related to VOCs in the cocoa infusion. The principal component analysis (PCA) began with the analysis of a matrix that describes the particle size (A, B, and C), the matrix (CS and CSI), and the functional groups of the VOCs (acids, alcohols, aldehydes, alkanes, esters, ketones, pyrazines, terpenes, terpenoids, and others).

## 3. Results and Discussion

### 3.1. Macro and Microelements

Among the potential beneficial effects of CS, the content of minerals (macro and microelements) represents an important feature of these ingredients. As for fibers and bioactive molecules, minerals are important nutrients for human wellbeing and represent another class of valuable compounds that can be recovered.

Three classes of elements were searched: macro-elements (calcium, magnesium, phosphorous, potassium, and sodium), micro-elements (copper, chromium, iron, manganese, nickel, and zinc), and other compounds (aluminium) (Table 1).

Scarce are the data relative to the mineral content of cocoa shells after roasting. In this regard, Raji Alex [25] reported in Nigeria (Forastero variety) values ten-fold lower than those reported in our study. In particular, they found values of calcium (0.0591 mg/g), potassium (0.187 mg/g), copper (0.00072 mg/g), zinc (0.0012 mg/g), iron (0.00542 mg/g), and magnesium (0.00572 mg/g). What comes out from the literature in respect of these last metals is that they mainly come from fertilization and the massive use of pesticides and fungicides [26], which increase their content in the soil. Aluminium and nickel are normally contained in vegetable sources, and their concentrations steeply increase due to human activities such as extraction, soil acidification, and water acidification [27,28]. A simple calculation performed just on two of the most abundant minerals traced shows that even a little inclusion of these by-products (1.5% addition) in food can increase the daily intake of calcium and potassium by about 0.00057 g for CS and 0.0038 g for CS.

A shared output is the fact that CS have great amounts of macro and microelements and can be used as integrators. Soetan et al. [29] remarked how humans are large consumers of minerals with high specificity for calcium and iron, which are highly used for bone renovation, the right functioning of blood’ red cells, and cellular respiration. Magnesium, copper, selenium, zinc, iron, manganese, and molybdenum are important enzyme co-factors. Conversely, some minerals are not involved in any biochemical pathway and can even be toxic; aluminum and nickel are among them [27,28]. Given the persistence of aluminum in the body, EFSA established a tolerable weekly intake TWI of 1 mg/kg bw/week. On the other hand, the values of nickel here found were lower than those regulated by the EFSA, which stabilized a tolerable daily intake (TDI) of 13 μg/kg bw. Thus, we can state that cacao shell can be used as an ingredient in low quantities.

The concentration of potassium (K), a macro-bio-element vital for a normal nervous system and to maintain water balance in the body, was determined to be between 13,800 mg/kg and 17,500 mg/kg.

### 3.2. Color Parameters and pH Values of CSI

Color parameters and pH values of infusion are shown in Table 2.

The color of the cocoa shell infusion showed a brown color similar to that found in some types of black tea. The L* parameter presented low values without statistically significant differences (*p* < 0.01) between the treatments. The effect of particle size was observed mainly in the parameters a* (+a indicates red, −a indicates green), b* (+b indicates yellow, −b indicates blue), and h*_ab_; as particle size reduced, a*, b*, and h*_ab_ also decreased significantly (*p* > 0.01), which suggests that the decrease in particle size increases the release of brown-colored compounds, especially melanoidins (red pigments) derived from the Maillard reaction and proanthocyanidins [30].

Similar results were obtained by Rojo-Poveda [12] in cocoa shell infusion (Forastero variety) with the French press maceration method, contrary to percolation extraction methods that manage to extract a more significant amount of compounds that increase the parameters a* and b*. The decrease in the h*_ab_ values (47.08 to 32.64) implies a fraction of the transition from the yellow (90°) to the red (0°), which corroborates that the decrease in particle size releases red pigments such as melanoidins. The CSI-B and CSI-C samples have a red hue (30.0–37.5), and the CSI-A has an orange-red hue (45.0–52.5).

Although the cocoa shell infusion was presented at a pH between 5.60 and 5.84 (Table 2), no effect on color was observed. It is known that some pigments found in cocoa, such as anthocyanins, can change their color according to pH.

For the CSI samples, particle sizes did not affect the visual perception since human vision cannot perceive ΔE* differences lower than 3 units.

**Figure 1:**
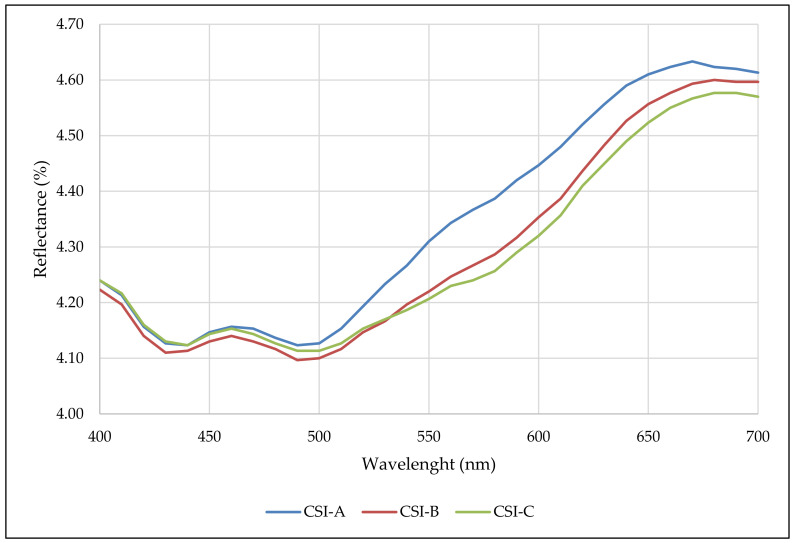
Reflectance spectra (400–700 nm) of the cacao shell infusion (CSI) for the three treatments.

The reflectance spectrum (400–700 nm) obtained from the cocoa shell infusion is also presented (Figure 1). From 500 nm on, a pronounced increase was observed, indicating a considerable contribution of photosynthetic pigments (yellow and orange pigments), phenolic compounds, and melanoidins (red pigments), also observed in cocoa shells [1]. Samples CSI-A, CSI-B, and CSI-C showed isosbestic points at wavelengths between 400–530 nm and 640–700 nm; only CSI-A showed isosbestic points at 540–630 nm. These samples showed higher values at these wavelengths than other samples.

From a reflectance point of view, particle sizes do not really influence most wavelengths (they are isosbestic); they were only affected at the largest particle sizes (>710 µm) at wavelengths 540–630 nm.

In general, the CSI color depends directly on the contribution of water-soluble pigments such as polyphenols, anthocyanins, and melanoidins derived from the fermentation, drying, and roasting processes (Maillard reactions). Therefore, it is possible through color studies to visualize the contribution of chemical fermentation indicators [31] and predict the shelf life of the product [32].

### 3.3. Microbiological Counts in Cocoa Shell and Cocoa Shell Infusion

It is well known that after fermentation and drying, cacao shells are contaminated with bacteria, yeast, and filamentous fungi that are eliminated during the roasting process (15 min, 135 °C). However, if the shells are separated from the nibs and storage procedures are not followed correctly, cross-contamination can occur. Table 3 shows the shell microbiota, which was contaminated prevalently by lactic acid bacteria (3.06 ± 0.55 Log CFU/g), mesophilic aerobic bacteria (4.85 ± 0.06 Log CFU/g), and yeast (3.34 ± 0.27 Log CFU/g). Total coliforms and molds were present in very low amounts. Many of these microorganisms can be thermo-, osmo-, and desiccation-tolerant and be present for a long time in the samples [33]. Although the microbial load found in the shell was low and complies with the recommendations for cocoa powder (NTC 518) [34], it may be necessary to incorporate an additional process to ensure the safety of the product without losing the functional characteristics of the beverage, especially in applications that do not use heat, such as iced tea. Non-thermal treatments can be an alternative to complement the thermal roasting process [35]; in the same way, controlled fermentations (starter cultures) can be another alternative to favor the presence of probiotic microorganisms in the shell [36], decreasing the populations of undesirable microorganisms.

Finally, no microorganisms were detected in the cocoa shell infusions, showing that the preparation temperature significantly decreased the initial load, which favors the consumer.

### 3.4. Ochratoxin A

The natural OTA content in the different cocoa shell and cocoa shell infusion samples was summarized in Table 4. As evidenced, OTA content was statistically different (*p* ≤ 0.05) in the sample CS-A (0.5314 ± 0.14) compared with CS-C (0.098 ± 0.03). However, there were no statistically significant differences (*p* ≥ 0.05) with CS-B (0.314 ± 0.23) samples, suggesting that the size of the particles directly correlates with the occurrence of ochratoxin A [37].

In the infusion, it was observed that almost all the ochratoxin A present in the cocoa shell passed into the infusion. In fact, the solubility of OTA in water is high (0.023 g/L).

There are no maximum permitted levels of mycotoxins in vegetable infusions. Therefore, as suggested by Caldeirao et al. [38], the exposure assessment was performed using the Margin of Exposure (MoE). The MoE is a tool to examine possible safety concerns arising from the presence of genotoxic and carcinogenic substances, such as OTA, in food and feed. As reported by EFSA [17], MoE values < 10,000 indicate a high risk of public health concern. Cocoa shell infusions presented values of this index above 10,000, which indicates a low potential health problem due to ochratoxin A. Although the values found for CSI-A and CSI-B are close to the umbral, the Panel on Contaminants in the Food Chain notes that this MoE is likely to be particularly conservative in this case since evidence of a direct interaction of OTA with DNA is inconclusive for the formation of renal tumors [17].

### 3.5. Biogenic Amine Content

To the best of our knowledge, no study has quantified biogenic amines (BAs) in cocoa shell infusions. Arlorio et al. [39] reported only the presence of tyramine ranging from 0.025 to 0.110 g L^−1^. In another study on tea infusions [40], very few contents of BAs were extracted and quantified, mainly polyamines (putrescine, spermine, and spermidine). Even in this case, low amounts of tyramine were detected.

Restuccia et al. [41] studied the BAs profile of cocoa-based items such as milk beverages, glazes, and spreads, both of organic rather than conventional provenience. Generally, organic cocoa-containing goods are poorer in BAs.

No BAs were detected in the CSI analyzed here. Aside from the scarcity of available literature, those data reported anyway refer to small amounts. We may hypothesize that the tuning of the extraction may somehow enhance the availability of BAs from CSI. In fact, as highlighted by Vinci and Maddaloni [42], in coffee and other nervine beverages that are not fermented, BAs are present. As an example, in coffee, differences between espresso and Turkish style are found. High-temperature employment limited the extraction of BAs with respect to coffee powder decoctions or instant coffees. Similarly, in tea, BAs are influenced by leaf provenience, processing, and flush; generally, green teas are richer in polyamines. A longer time of infusion, higher quantities of CS, and leaving the by-product boiling in water may increase the extraction.

### 3.6. Antioxidant Capacity, Total Phenolic Compounds, and Polyphenol Profile

The antioxidant activity determined by two different methodologies (ABTS and DPPH) and the total phenolic compounds (TPC) of the infusion showed a statistically significant dependence (*p* < 0.05) on the CS particle size. Thus, the infusion prepared with the smallest particles presented a greater antioxidant capacity (Table 5). This behavior can be attributed to smaller particle sizes having a greater exposed surface area, which leads to a more significant release of bioactive compounds with antioxidant properties, including polyphenols, into the infusion. These results agree with what was reported by Botella-Martínez [43] in cocoa bean shell flours, who obtained a similar trend.

Some studies have been carried out showing the antioxidant capacity of the CS using different extraction methods (DPPH 2.35–5.53 and ABTS 3.39–11.55 mg TE g^−1^), which present lower values of antioxidant activity in other varieties of cocoa [43]. The antioxidant activity and TPC found are similar to those reported for herbal teabags (mixtures of herbs) [44], rosehip tea beverages [45], cocoa bean shell and black tea [46], and cocoa bean shell (criollo variety) [13]. In general, criollo variety cocoa is characterized by having high values of antioxidant activity [18].

**Figure 2:**
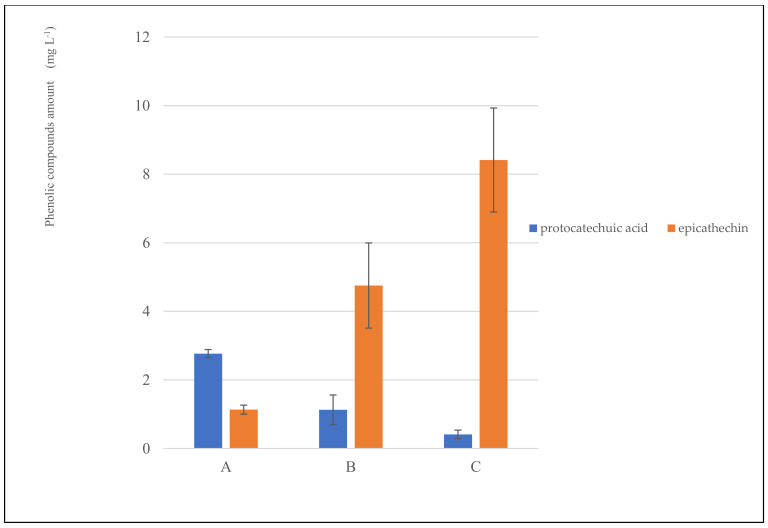
Polyphenolic profile of CSI obtained from different particle sizes (CSI−A, > 710 µm; CSI−B, > 425 < 710 µm; CSI−C, < 425 µm).

The antioxidant properties of cocoa and its co-products can be attributed to the high content of bioactive compounds such as catechin, epicatechin, isoquercetin, theobromine, caffeine, melanoidins, and proanthocyanidins [30]. Only epicatechin and pro-tocatechuic acid, highly soluble in water, were detected in the cocoa infusion (Figure 2). The linear correlation coefficient was calculated. Epicatechin was positively correlated with antioxidant activity (ABTS r = 0.9602 and DPPH r = 0.9923) and total phenolic compounds (r= 0.9886). On the contrary, protocatechuic acid was negatively correlated with antioxidant activity (ABTS r = −0.9984 and DPPH r = −0.9948) and total phenolic compounds (r = −0.9972). Catechin can represent about 37% of the total polyphenols in cocoa. In comparison, protocatechuic acid belongs to the group of anthocyanins that can represent 4% of the total polyphenols in cocoa.

The values of antioxidant activity and TPC found allow us to conclude that the fraction of biomolecules with antioxidant activity that passes into the infusion is favored by the decrease in particle size. The reduction in particle size becomes an important step to increase the extraction during CSI preparation so that the consumer can obtain more significant benefits from incorporating these compounds into their diet [13].

### 3.7. Organic Acids and Sugar Content

During the fermentation process, the microorganisms metabolize the sugars available in the mucilage and produce alcohols, organic acids, and other biomolecules that can remain in the cocoa shell, degrade, or volatilize during the same fermentation, drying, or roasting. It has been reported that these compounds are highly variable and depend on the cocoa variety and the fermentation and drying conditions [47]. Table 6 shows the content of the CSI of organic acids, sugars, and glycerol.

Our results show that the sugars (fructose, glucose, and saccharose) and organic acids in the CSI are present in low concentrations. The results found in the infusion are much lower than those reported in the cocoa shell using ortho-phosphoric acid extraction [43] and subcritical water extraction [48], which indicates that the extraction conditions used in the preparation of the infusion fail to extract all organic acids, sugars, and alcohols present in the cocoa shell.

The particle size did not present statistically significant differences between the treatments (*p* > 0.05). This may indicate that the infusion preparation conditions only manage to extract the organic acids and sugars found on the surface of the shell since, after the decrease in particle size, an increase in their solubility was not observed. There needs to be data in the literature on the concentrations of sugars and organic acids in the cocoa shell infusion.

### 3.8. Volatile Organic Compounds (VOCs)

**Figure 3:**
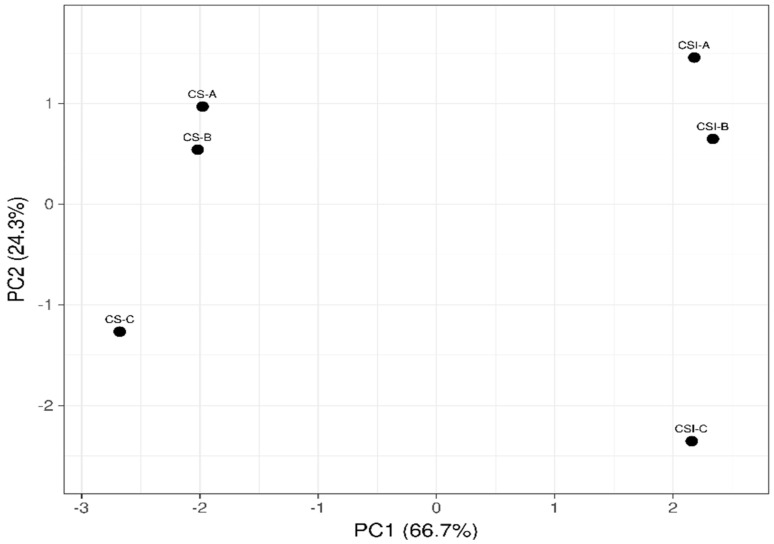
Principal component analysis related to the COVs in cocoa shells (CS) and cocoa shell infusions (CSI) with different particle sizes (CSI−A, >710 µm; CSI−B, >425 <710 µm; CSI−C, <425 µm).

A principal component analysis (PCA) was carried out to demonstrate the influence of the treatments on the VOCs. Figure 3 shows the distribution of the variables analyzed in the two first principal components. As for PC1 (66.7%), this component was influenced by the matrix; the VOCs of the infusion were on the positive axis, while those of the CS were on the negative axis. A separation of four units was observed between the VOCs of the CS and those of the infusion. This indicates that the VOCs vary by changing from a solid matrix to an aqueous matrix, with the solubility of the components in water having a notable influence. Regarding PCA2 (24.3%), particle size influenced this component. The particle sizes A and B (close) were located on the positive axis. In contrast, on the negative axis, particle size C was located. This indicates that particle sizes A and B presented more remarkable similarities in the VOCs and differed from particle size C. For powders, particle size affects bulk density and strongly influences the phenomenon of the transportation of molecules in the matrix. Numerous studies have shown that the particle characteristics in terms of size, shape, and structure, flowability, and foam properties such as the foam’s size and stability influence the sensory performance of the powders used for beverage infusion preparation [49]; this impacts both the taste and the aroma of the product. Huang et al. [50] observed that particle size and surface morphology analyses showed differences in the physical properties of the matcha, which translated to variations in the release and stability of chemicals (non-volatile and volatile compounds) and sensory perception (richness). The release of volatile and non-volatile compounds in the liquid matrix could be increased or decreased, depending on the preparation method. Espresso, for instance, uses a very fine coffee powder, while filter brewing preparations use larger particle sizes [51]. Our data demonstrated that the reduction of particle size increases the surface area and therefore releases more VOCs. This effect occurs both in a solid matrix (CS powder) and in a liquid matrix (CS infusion).

Forty-five compounds were identified in cocoa shell and forty-two in cocoa shell infusion. They are shown by the functional chemical group in Table 7. These compounds are developed during the cocoa’s fermentation, drying, or roasting. According to the processing conditions, pleasant or unpleasant aromas can be generated in the final product, which is why they are considered quality parameters.

In general, the most representative classes of compounds in CS were acids (18.2–41.8%), aldehydes (15.2–23.4%), pyrazines (12.8–23.1%), alcohols (9.7–11.9%), esters (6.3- 9.7%), and terpenes and terpenoids (5.3–5.9%). It was found that the decrease in particle size produces changes in the relative percentage area of VOCs. It was observed that the reduction in particle size causes a decrease in the relative area of alcohols, aldehydes, alkanes, esters, ketones, and pyrazines. At the same time, terpenes and acids increased, especially acetic acid.

Few studies have been carried out on VOCs in cocoa shells, so comparisons and discussions are difficult to carry out, even more so when it has been shown that the variability of VOCs in cocoa beans depends on multiple factors such as pod storage and bean roasting temperature [52], fermentation time [53], turning, pod storage, and fermentation time [54], which also affect the VOCs of cocoa shells. Barbosa-Pereira et al. [55] conducted a study with cocoa bean shells (<250 µm) from different cultivars and geographical regions, although the cocoa shell of the Criollo variety from Colombia was not included. Our results are comparable with those obtained in Criollo and Nacional cultivars that display, on average, higher amounts of pyrazines, acids, alcohols, and ketones.

Regarding the infusion, the most representative classes of compounds were aldehydes (48–51.2%), esters (11.8–16.0%), ketones (6.7–9.5%), and terpenes and terpenoids (3.9–9.8%). Only terpenes and terpenoids retained the trend of increasing relative amounts with decreasing particle size. The CSI-A presented higher relative amounts of acids, aldehydes, and alkanes; the CSI-B ketones and esters; and the CSI-C alcohols and terpenes. Regarding the effect of preparing the infusion, it was observed that the nonpolar VOCs were expressed in greater relative quantity, such as nonanal (36.5–38.3%), 2-nonanone (5.4–7.4%), or linalool (3.0–3.8%). As expected, the interaction of polar VOCs with water through the formation of hydrogen bonds or dipole-dipole interactions favors the solubility of these compounds, decreasing their volatilization. This also allows nonpolar compounds to increase in volatility and be expressed in greater quantity. Additionally, interactions between VOCs and non-volatile soluble molecules such as salts and peptides may occur, which may favor or disfavor the solubility of some specific VOCs [56].

Of the organic acids found, acetic acid is the main volatile compound in CS. It comes mainly from the fermentation and drying stages [57] and is expressed in higher relative concentrations in the smaller CS. However, the relative concentration of acetic acid in CSI was low, with no differences between the treatments, corroborated with the concentration found by HPLC (Table 5) and the pH in CSI (5.60–5.84). Acetic acid is related to vinegar’s sour and astringent tastes. Cis-9-octadecenoic acid (oleic acid) was found in CS and CSI. This fatty acid provides a fat aroma. These long-chain fatty acids are present due to the parts of the grain that remain in the CS [3].

Other acids were found only in CS, which can contribute unpleasant aromas but could not be expressed in the CSI aroma, such as isovaleric acid (stale cheese aroma) and nonanoic acid (rancid aroma) that are presented as indicators of inadequate fermentation, and 2-methylbutanoic acid (stale cheese aroma). 4-hydroxybutanoic acid was also found, which comes from the fermentation stage and is common in fermented beverages.

Most of these acids are generally found in cocoa-derived products [51]. Based on the substantial concentrations and odor activities of most volatile acids, they are expected to contribute to the predominant notes of acid and vinegary flavor in CS but have not manifested themselves in the aroma of CSI.

Of the five alcohols found in the CS, phenyl ethyl alcohol comes mainly from the pulp (a pleasant floral smell), and 2,3-butanediol, produced by fermentation of microorganisms and found naturally in cocoa butter, were the ones that presented the highest relative amounts in the CS. In the infusion, the main alcohol was 2-heptanol, found among the volatile compounds of cocoa pulp, bean [53,58], and shell [55]. 2-heptanol decreases with the progress of fermentation and drying but increases with roasting temperature [51]. It has floral and sweet notes such as citrus, fruity, lemongrass, fresh, herbal, and green. The alcohols together give the infusion pleasant fruity odors. No trend dependent on particle size was found in CSI, possibly due to the high solubility of alcohols.

Six aldehydes were found in the CS. Nonanal was the major component; it decreases with the decrease in particle size (18.5–11.7%); it is found naturally in the cocoa pulp and in the shell [55], but it was not found in cocoa liquors [52]. In the infusion, aldehydes represent the majority group (51.2–48%), of which 38.3–36.5% correspond to nonanal, contributing a fragrant, woody-like aroma. The decanal (4.1–2.3%) is the second-largest aldehyde; it confers sweet, orange, and waxy notes. In general, aldehydes with very odorous compounds give the infusion pleasant notes.

Three ketones were found in the CS. They were found in a higher proportion in CS-B and a lower proportion in CS-C. 2-nonanone was the major component (2.6–1.3%). The same trend was found in the infusion, with a high expression in the aroma of 2-nonane, conferring attractive green, weedy, and herbal nuances.

Twelve esters were found in the CS, which decreased with particle size. Fatty acid esters were mainly expressed. The main component was acetic acid-2-phenylethyl ester (5.3–1.9%), which gives the rose and honey scent. This ester has been found in cocoa liquor and is favored by increasing the roasting temperature [52]. In the infusion, the percentage of esters increased; the acid-2-phenylethyl ester was also the major component.

Two important pyrazines were found in defining the CS aroma: trimethylpyrazine (2.0–1.6%) and tetramethylpyrazine (21.2–11.2%). These pyrazines are responsible for the aroma of roasted chocolate; they are found in cocoa beans [54], cocoa shells [55], and even cocoa liquor [52]. The relative amount decreased in the infusion due to the solubility of pyrazines in water, so only tetramethylpyrazine was found, presenting a higher concentration in CS-B and CSI-B.

Two terpenes were found for the CS aroma: linalool and α-limonene; they also contribute to fine cocoa’s fruity and floral odors. Linalool has been found in Forastero cocoa liquor [52] and Forastero cocoa shell [55]. While α-limonene is better expressed in Criollo and Trinitario cacao [59], its relative quantity increases significantly with the decrease in particle size.

Although the aroma sensation is due to the integration of all the volatile components, the aroma of the cocoa infusion stands out due to the presence of nonanal, 2-nonanone, tetramethylpyrazine, α-limonene, and linalool, which present few variations by particle size effect, except limonene, which is in a higher proportion in CSI-C.

### 3.9. Sensory Analysis Quantitative Descriptive Analysis (QDA)

A panel test for the evaluation of color, odor, and taste was performed on a total of six samples divided into two triplets. The first triplet identified here as A, B, and C comprises the infusions of each particle size tasted at 55 °C, while triplets D, E, and F comprise the same infusions at 12 °C. This approach was pursued because of the versatility of the infusion on different occasions. To better understand the perception of aromatic profiles, panelists were asked to discriminate odors by sniffing samples (odors) and after drinking each of them (retro-nasal). As expected, hot infusions resulted in a general increase in value for all descriptors, especially chocolate-like and aromatic in the retro-nasal odor class. CS is, in fact, a highly aromatic ingredient that delivers many of the attributes of cocoa and chocolate.

As recently reported by San Siow et al. [11], these characteristics are due to the fermentation and roasting processes, which increase the content of aldehydes and ketones typical of dark chocolate. The same authors also explained how the fermentation step of cocoa beans adds sweetness to the final infusions.

**Figure 4:**
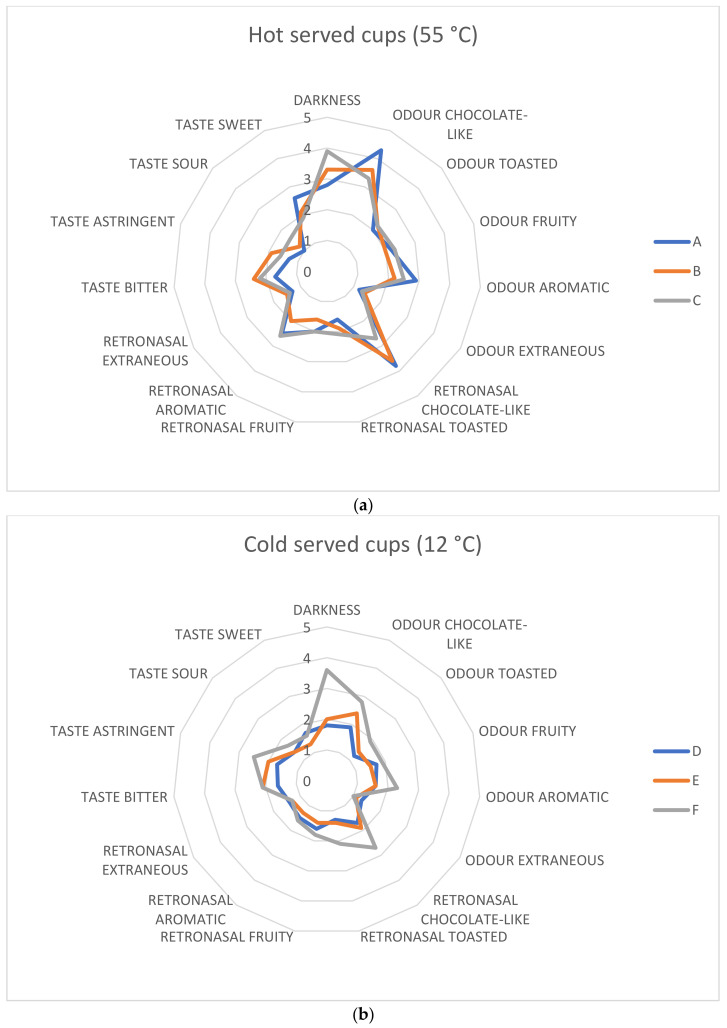
Spider plots of hot (**a**) and cold (**b**) infusions obtained from different particle sizes (A and D, >710 µm; B and E, >425 <710 µm; C and F, <425 µm). Mean values of scores for descriptors are reported.

A significant effect (*p* < 0.05) both of the particle size and of the temperature of tasting was found on some sensory attributes of cocoa shell infusion. Particle size influenced mainly the color intensity (expressed as darkness) of all samples, no matter the temperature of degustation, increasing its value. For hot infusions, panelists have noted an increased bitterness, sourness, and astringency given by smaller particle sizes. From the spider plots reported in Figure 4 (a, hot samples; b, cold samples), it seems that reduced sizes (F) contributed to the perceived attributes of cold cups, while medium and large particles scored lower. Conversely, hot infusions received significantly (*p* < 0.05) higher scores for larger grinding diameters (A), in particular for “positive” and typical descriptors. Reducing the particle size had an impact mainly on the taste class of descriptors, characterizing samples B and C for significantly (*p* < 0.05) increased bitterness, astringency, reduced sweetness, and sourness in comparison with sample A. This trend is confirmed both in hot and cold samples. In a deep study conducted on coffee quality evaluation by Chapko and See [60], it was found that temperature affects the perception of aromas. For taste, specific comments must be made. Authors have found a non-linear trend for bitterness in respect of temperature of serving (it slowly decreases from 70 °C to 40 °C and increases at 25 °C) and a linear trend for sourness (its perception increases from hot to cold values). These results led to the conclusion that bitterness is not influenced by temperature, while sourness is significantly enhanced by decreasing the temperature of the serving. Of course, coffee is different from the infusion here proposed, but somehow we found that sourness was higher in cold samples. We have registered, as said, even increased bitterness and astringency in cold samples and reduced sweetness. The piece of information we can add to this study is that, in all cases, reduced particle size augments the perception of tastes, with the exception of sweetness. This is likely due to the increased extraction rate assured by the higher volume/surface ratio per time. For what concerns odors and aromas, aside from the general consideration of the temperature effect on the volatile phase of all matrices, we observed that reduced particle size has increased the perception of these classes, especially in cold samples. For teas, it was seen that diminishing particle size negatively influences the release of antioxidant species, contributing to the final bitterness and astringency and so reducing the overall acceptability [61]. On the whole, smaller particle sizes might be useful to gather aromaticity in cold-served infusions, but burdens for the extraction of bitter and astringent compounds must be set. Designing a cold extraction from small particle sizes could be a future step in this study.

## 4. Conclusions

The cocoa shell infusion represents a safe drink for the consumer and an opportunity to consume biomolecules of interest for health. It is safe because no microorganisms, biogenic amines, or low content of ochratoxin A were found, which does not represent a potential health problem. Emphasizing that adequate process and hygiene conditions must be maintained to ensure the safety of cocoa throughout the process until the CS is obtained. Likewise, the cocoa shell infusion provides macro- and micronutrients and water-soluble pigments such as polyphenols, anthocyanins, and melanoidins derived from the fermentation, drying, and roasting processes. The decrease in particle size increased the antioxidant capacity and the polyphenol content, especially epicatechin, which represents for the consumer a greater availability of biomolecules but also a greater perception of bitter and astringent flavors.

## Figures and Tables

**Table 1:** Mineral composition (mg g^−1^) of cocoa shell.

Elements	Concentration (mg/g)
Macrominerals	
Calcium	0.38 ± 0.05
Magnesium	0.42 ± 0.04
Phosphorus	0.36 ± 0.05
Potassium	2.53 ± 0.25
Sodium	0.03 ± 0.008
Microminerals	
Copper	0.025 ± 0.002
Chromium	0.0023 ± 0.0002
Iron	0.03 ± 0.02
Manganese	0.023 ± 0.0027
Nickel	0.017 ± 0.0021
Zinc	0.045 ± 0.01
Other elements	
Aluminium	0.02 ± 0.02

Results are expressed as means ± standard deviations.

**Table 2:** Color parameters and pH values of cocoa shell infusion.

Infusion	L*	a*	b*	h*_ab_	ΔE*	pH
CSI-A	24.67 ± 0.17 a	0.84 ± 0.02 b	0.90 ± 0.03 c	47.08 ± 0.35 c		5.84 ± 0.05 b
CSI-B	24.44 ± 0.02 a	0.82 ± 0.01 b	0.61 ± 0.01 b	36.80 ± 0.57 b	0.37 ± 0.01	5.60 ± 0.09 a
CSI-C	24.40 ± 0.09 a	0.73 ± 0.02 a	0.47 ± 0.03 a	32.64 ± 1.02 a	0.52 ± 0.07	5.80 ± 0.02 b

Results are expressed as means ± standard deviations. Different letters in the same column indicate significant differences (*p* < 0.01).

**Table 3:** Microbial load in cocoa shell (CS) and cocoa shell infusion (CSI).

	Microbial Counts					
	Cocoa Shell (Log CFU/g CS)			Cocoa Shell Infusion (Log CFU/g CSI)		
	CS-A	CS-B	CS-C	CSI-A	CSI-B	CSI-C
Lactic acid bacteria	3.06 ± 0.55	2.07 ± 0.46	2.52 ± 0.53	n.d	n.d	n.d
Acetic acid bacteria	<1.0	<1.0	<1.0	n.d	n.d	n.d
Mesophilic aerobic bacteria	4.85 ± 0.06	3.90 ± 0.06	4.09 ± 0.12	n.d	n.d	n.d
Total coliforms	1.21 ± 0.19	1.69 ± 0.25	<1.0	n.d	n.d	n.d
Moulds	1.20 ± 0.17	<1.0	<1.0	n.d	n.d	n.d
Yeast	3.34 ± 0.27	2.0 ± 0.0	2.0 ± 0.0	n.d	n.d	n.d

Results are expressed as means of three samples ± standard deviations. n.d.: no detected.

**Table 4:** Comparison of OTA occurrence in cocoa shell and cocoa shell infusion samples and risk characterization of OTA through determination of Margin of Exposure (MoE).

Sample	Ochratoxin A [μg kg^−1^] Cocoa Shell	Ochratoxin A Infusion [μg kg^−1^] Cocoa Shell Infusion	MoE
A	0.5314 ± 0.14 b	0.366 ± 0.44 b	23,324.0
B	0.3415 ± 0.23 ab	0.568 ± 0.32 b	15,042.4
C	0.0988 ± 0.03 a	0.099 ± 0.02 a	86,228.1

Different letters indicate significant differences (*p* < 0.05; Tukey HSD post-hoc test) between the samples. For characterization of chronic neoplastic effects, an MoE of ≥ 10,000 between the selected neoplastic reference point and calculated exposures would be of low health concern.

**Table 5:** Antioxidant capacity and total phenolic compounds of cocoa shell infusions.

	Antioxidant Capacity		Total Phenolic Compounds
	µmol TE/100 mL		mg GAE/100 mL
Cocoa Shell Infusion	ABTS	DPPH	TPC
CSI-A	134.06 ± 4.73 a	172.12 ± 5.16 a	19.94 ± 1.51 a
CSI-B	160.68 ± 5.37 b	194.36 ± 17.21 b	30.96 ± 2.25 b
CSI-C	169.61 ± 6.93 c	208.86 ± 20.87 b	37.46 ± 2.71 c

Legend: The results were established using the infusion as the extraction medium. Infusion: 1 g of cacao shell in 100 mL of water at 96 °C for 5 min. TE: Trolox equivalent; GAE: Galic acid equivalent. Results are expressed as means ± standard deviations. Different letters in the same column indicate significant differences (*p* < 0.05).

**Table 6:** Organic acids and sugar content (g L^−1^) in cocoa shell infusion.

Cocoa Shell Infusion	Acetic Acid	Succinic Acid	Fructose	Glucose	Saccharose	Glycerol
CSI-A	0.049 ± 0.009	0.015 ± 0.006	0.024 ± 0.007	0.494 ± 0.36	0.641 ± 0.35	0.016 ± 0.004
CSI-B	0.046 ± 0.016	0.012 ± 0.001	0.030 ± 0.004	0.488 ± 0.32	0.501 ± 0.31	0.017 ± 0.005
CSI-C	0.037 ± 0.009	0.012 ± 0.001	0.045 ± 0.028	0.494 ± 0.33	0.499 ± 0.30	0.017 ± 0.005

Results are expressed as means ± standard deviations. No significant differences were found (*p* > 0.05).

**Table 7:** Relative percentage area of COVs in cocoa shell and cocoa shell infusion of samples at different particle sizes.

		Cocoa Shell			Cocoa Shell Infusion		
Component	RT	CS-A	CS-B	CS-C	CSI-A	CSI-B	CSI-C
**Acids**							
Acetic acid	3.36	15.9	15.0	37.4	0.7	0.5	0.5
Isovaleric acid	8.762	1.1	1.0	1.4	n.d	n.d	n.d
2-Methylbutanoic acid	9.037	0.9	1.1	2.1	n.d	n.d	n.d
4-Hydroxybutanoic acid	9.462	0.0	0.0	0.3	n.d	n.d	n.d
cis-9-Octadecenoic acid	14.204	0.5	0.5	0.3	0.6	0.7	0.6
Nonanoic acid	15.66	0.7	0.6	0.3	n.d	n.d	n.d
Subtotal		19.1	18.2	41.8	1.3	1.2	1.1
**Alcohols**							
2,3-Butanediol	6.421	1.7	1.7	4.1	n.d	n.d	n.d
2-Heptanol	9.302	0.8	0.7	1.3	2.3	2.0	2.6
3,3-dimethyl-1-butanol	10.948	0.0	0.0	0.4	0.4	0.5	0.5
Phenylethyl Alcohol	13.384	9.4	8.4	3.8	0.8	0.8	0.6
5,9-dimethyl-1-decanol		n.d	n.d	n.d	0.7	0.6	0.7
3-Methylcyclopentanol	16.49	0.0	0.1	0.1	0.4	0.2	0.4
Subtotal		11.9	10.9	9.7	4.6	4.1	4.7
**Aldehydes**							
2-Oxopropanal	9.992	0.7	0.6	1.0	0.1	0.1	0.3
Benzaldehyde	10.598	1.0	1.0	0.6	1.4	1.3	2.6
2,4-Nonadienal		n.d	n.d	n.d	1.3	0.9	0.9
Octanal		n.d	n.d	n.d	2.4	1.9	2.2
Benzeneacetaldehyde	12.183	1.4	1.2	1.0	2.7	3.5	3.5
Nonanal	13.214	18.5	17.1	11.7	38.3	36.7	36.5
Decanal	14.839	1.7	1.5	0.8	4.1	2.7	2.3
2-Methylpentanal	16.105	0.0	0.1	0.1	1.0	0.8	0.5
Subtotal		23.4	21.7	15.2	51.2	48.0	48.8
**Alkanes**							
Z,Z,Z-4,6,9-Nonadecatriene	12.418	1.2	1.1	0.7	0.6	1.2	0.4
3-Ethyl-5-(2-ethylbutyl)-octadecane	14.729	1.9	1.5	0.5	1.4	0.9	0.6
2-Methyldecane	17.55	0.5	0.4	0.0	1.9	1.0	0.4
Subtotal		3.6	3.1	1.2	3.8	3.2	1.4
**Esters**							
Acetic acid, pentyl ester		n.d	n.d	n.d	2.1	1.9	0.4
2-Heptanol, acetate	12.033	1.0	0.9	0.6	1.5	2.3	2.1
Z,Z,Z-8,11,14-Eicosatrienoic acid, methyl ester	12.303	0.6	0.6	0.5	n.d	n.d	n.d
9-Octadecen-12-ynoic acid, methyl ester	12.668	0.5	0.5	0.6	1.1	1.4	1.2
Hexadecanoic acid, ethyl ester	14.654	0.7	0.7	0.3	0.7	0.9	0.7
Butanoic acid, 2-ethyl-, 1,2,3-propanetriyl ester	15.024	0.9	0.9	0.4	n.d	n.d	n.d
9-Octadecen-12-ynoic acid, methyl ester	15.47	0.0	0.0	1.3	n.d	n.d	n.d
Acetic acid, 2-phenylethyl ester	15.585	5.3	4.9	1.9	3.9	4.7	3.7
2-Propenoic acid, 4-methylpentyl ester	16.6	0.1	0.1	0.1	0.4	0.0	0.4
Dodecanoic acid, ethyl ester	17.5	0.2	0.3	0.0	0.5	0.4	0.5
Octadecanoic acid, (2-phenyl-1,3-dioxolan-4-yl)methyl ester, cis-		n.d	n.d	n.d	1.0	1.1	0.6
Valeric acid, 3-tridecyl ester	21.067	0.1	0.1	0.1	1.0	0.6	0.3
Hexadecanoic acid, 1-(hydroxymethyl)-1,2-ethanediyl ester		n.d	n.d	n.d	1.6	1.8	0.5
Linolenic acid, 2-hydroxy-1-(hydroxymethyl)ethyl ester (Z,Z,Z)-	25.559	0.1	0.1	0.1	0.3	0.6	0.5
9,12-Octadecadienoic acid (Z,Z)-, 2,3-dihydroxypropyl ester	26.904	0.1	0.1	0.2	1.1	0.5	0.8
Subtotal		9.7	9.1	6.3	15.1	16.0	11.8
**Ketones**							
3-Hydroxy-2-butanone	4.215	0.5	0.5	1.1	n.d	n.d	n.d
2-Heptanone		n.d	n.d	n.d	1.7	1.3	0.6
2-Nonanone	12.964	2.3	2.6	1.3	5.8	7.4	5.4
4-hydroxy-2-methyl-3-phenyl-2-cyclopenten-1-one	15.825	0.5	0.5	0.3	0.7	0.7	0.6
Subtotal		3.3	3.6	2.7	8.2	9.5	6.7
**Pirazines**							
Trimethylpyrazine	11.393	2.0	1.9	1.6	n.d	n.d	n.d
Tetramethylpyrazine	12.894	17.4	21.2	11.2	4.0	4.9	4.5
Subtotal		19.5	23.1	12.8	4.0	4.9	4.5
**Terpenes and terpenoids**							
α-Limonene	11.903	4.0	4.0	4.2	0.9	2.5	6.1
Linalool	13.139	1.2	1.7	1.7	3.0	3.8	3.7
Subtotal		5.3	5.6	5.9	3.9	6.3	9.8
**Others**							
methoxy-phenyloxime		n.d	n.d	n.d	1.1	0.3	1.8
(3-Methyl-oxiran-2-yl)-methanol	9.852	0.9	1.0	2.0	n.d	n.d	n.d
Ethylenimine	11.018	0.4	0.3	0.4	0.9	0.9	0.4
Ethyl iso-allocholate	15.204	0.4	0.4	0.3	0.5	0.4	0.5
Phenethylamine	15.399	1.3	1.2	0.3	0.6	0.7	0.7
3,7-dimethyl-1-octene	18.221	0.1	0.1	0.1	1.3	0.7	0.4
1-Butanamine	20.381	0.0	0.1	0.1	0.6	0.6	0.5
Not identified		1.2	1.5	1.3	2.9	3.2	6.8
Subtotal		4.3	4.7	4.5	7.9	6.8	11.1

Particle sizes: CS−A and CSI−A (>710 µm); CS−B and CSI−B (>425 <710 µm); CS−C and CSI−C (<425 µm). n.d.: no detected.

## Data Availability

The data presented in this study are available on request from the corresponding author.
